# Original and Low-Cost ADS-B System to Fulfill Air Traffic Safety Obligations during High Power LIDAR Operation

**DOI:** 10.3390/s23062899

**Published:** 2023-03-07

**Authors:** Frédéric Peyrin, Patrick Fréville, Nadège Montoux, Jean-Luc Baray

**Affiliations:** 1 Université Clermont Auvergne, CNRS, Observatoire de Physique du Globle de Clermont Ferrand, UAR 833, F-63000 Clermont–Ferrand, France; 2 Université Clermont Auvergne, CNRS, Laboratoire de Météorologie Physique, UMR 6016, F-63000 Clermont–Ferrand, France

**Keywords:** ADS-B, LIDAR, air safety obligation

## Abstract

LIDAR is an atmospheric sounding instrument based on the use of high-power lasers. The use of these lasers involves fulfilling obligations with respect to air safety. In this article, we present a low-cost air traffic surveillance solution integrated into an automated operating system for the Rayleigh-Mie-Raman LIDAR of Clermont Ferrand and the statistical elements of its application over more than two years of operation from September 2019 to March 2022. Air traffic surveillance that includes the possibility of shutting off lasers is required by international regulations because LIDAR is equipped with a class four laser that presents potential dangers to aircraft flying overhead. The original system presented in this article is based on software-defined radio. ADS-B transponder frames are analyzed in real-time, and laser emission is stopped during LIDAR operation when an aircraft is detected within a 2 km radius around the LIDAR. The system was accredited in 2019 by the French air traffic authorities. Laser shutdowns due to the detection of aircraft near the Clermont Ferrand LIDAR caused a data loss rate of less than 2% during the period of application.

## 1. Introduction

The climate change we are currently facing makes crucial the need to maintain regular and long-term observations of the atmosphere and, more specifically, essential climatic variables such as water vapor, clouds and aerosols [1]. The in situ measurement of these variables at the surface of the earth is important, but knowledge of their vertical distribution across the whole troposphere is necessary to apprehend the physico-chemical, micro-physical and dynamical processes that influence their concentration in all their complexity. 

LIDAR (light detection and ranging) is a remote-sensing technique developed in the 1960s that allows the measurement of these respective vertical distributions with a high spatio-temporal resolution [2]. The principle of LIDAR is based on the emission of a vertical laser beam into the atmosphere whose light is backscattered by particles and molecules, collected by a telescope and analyzed using an optical and electronic device. The inversion of the backscattered signal provides vertical profiles of the atmospheric constituents. The Cézeaux-Aulnat-Opme-Puy-De-Dôme CO-PDD instrumented site [3] is part of the Observatoire de Physique de Clermont-Ferrand (OPGC). It is instrumented with COPLid (CO-PDD LIDAR) which uses a high-power laser to obtain, for example, vertical profiles of aerosol coefficients for the characterization of major volcanic [4] and biomass burning [5] plumes.

COPLid emits three wavelengths of 1064 nm, 532 nm and 355 nm. Regarding eye safety, the most critical wavelength is 532 nm, corresponding to the color to which the human eye has the greatest sensitivity. The 60825-1 European standard provides information on whether a laser beam is eye-safe in relation to the distance from the laser source. Moreover, the laser beam being vertical, the risk zone can be properly characterized: the eye-safe distance of the COPLid laser is 80 km from the laser source. As it stands, there is a potential danger to aviation safety.

Furthermore, regarding air traffic, operating LIDAR systems equipped with powerful lasers in IR, visible and UV wavelength ranges also requires compliance with international air traffic regulations, based on the Manual on Laser Transmitters and Flight Safety [6].

Consequently, LIDAR operators must at least wear protective glasses if they are working in the vicinity of the beam and furthermore, a safety system must be implemented to protect aircraft pilots who are passing through the beam. 

It is imperative that the LIDAR system is equipped with a device that makes it possible to eliminate ocular risk during the passage of an aircraft near the radiation. The principle of this device is to eliminate exposure to this hazard by evaluating potential risks, identifying air traffic and consequently enabling laser emission.

Usually, a supporting conventional radar system is implemented to determine the presence of an aircraft in the vicinity of the LIDAR airspace exploration zone. This radar makes it possible to establish the presence (position, distance, and speed) of an aircraft in the LIDAR firing zone and to intervene based on the measurement protocol to suspend laser emission until the aircraft passes and clears the area. However, the radar system remains costly, is potentially complex to implement and requires administrative authorization for radiofrequency (RF) emission, which is not always possible or easy to obtain, as a function of the site location. 

Alternatively, the International Air Traffic Association (IATA) has promoted the ADS-B system (automatic dependent surveillance-broadcast) in order to strengthen traffic control. This new concept is a real-time surveillance system used for air traffic surveillance [7], which makes it possible to know the position of aircraft constantly. ADS-B is a means of surveillance and airspace control recognized by the ICAO (International Civil Aviation Organization). Thanks to this system, aircraft transmit useful information, especially their identity and position, to any listeners equipped with an ADS-B receiver device, whether they are officials or individuals.

Therefore, as a complete and new alternative to radar, the use of an ADS-B system can make it possible to locate aircraft close to our operating LIDAR for air safety purposes. Therefore, thanks to the advances in radio frequency systems such as software defined radio (SDR), we decided to develop a solution based on this principle allowing us to receive and analyze useful information and to switch off the laser if needed.

## 2. Materials and Methods

### 2.1. Overview of COPLid

The LIDAR system operated at Clermont Ferrand from 2008 to 2022 was a mono-wavelength Rayleigh-Mie-Raman LIDAR, which provides continuous vertical tropospheric aerosol, cirrus and water vapor mixing ratios [8]. The emitted radiation at 355 nm was eye safe in respect of the 60825-1 European standard. Therefore, no surveillance system was necessary to emit this radiation [6]. After the first phase of LIDAR automation, the system was recently upgraded to a three-wavelength LIDAR based on an INDI Spectra Physics laser. This laser emits radiations at 1064 nm, 532 nm and 355 nm with a pulse repetition rate of 10 Hz. After beam expansion, the divergence of the laser beam in the atmosphere is 0.1 mrad.

Regarding the receiver, the wavelength splitting unit (WSU) has been completely redesigned in order to receive three new channels: 532 nm with parallel and perpendicular polarization separation, and a 1064 nm channel. A rotating stage allows the rotation of the WSU for polarization calibration [9].

For thermal stability and ergonomic reasons, COPLid was installed in a room with a hatch on the roof. A new supervisor LabVIEW software manages all the LIDAR measurement operations without any attendance. It manages acquisition control, the safety of the instrument (wind/rain sensors to open/close the hatch), the safety of the staff (intruder detection) and the safety of the air traffic (ADS-B system).

### 2.2. Overview of the ADS-B System

Advances in radio frequency (RF) systems such as software defined radio (SDR) allow surveillance of the airspace around the LIDAR. Furthermore, SDR is a system that favors software signal processing over the hardware usually used in conventional RF systems. An electronic system based on the principle of SDR permanently receives the ADS-B signal emitted by the aircraft on the 1090 MHz frequency. There are different types of ADS-B link available including the 1090ES (“1090 MHz Extended Squitter”) that we use. It is an extension of the MODE-S radar transponders, which transmit on 1090 MHz. On airplanes equipped with MODE-S and TCAS (Traffic Collision Avoidance System), these transponders already make it possible to send and receive 56-bit messages. A modification allows them to send 112-bit messages, which is sufficient for ADS-B. 

On the ground, ADS-B information can be received either by a MODE-S radar or by a simple omnidirectional antenna, which is much less expensive. The captured frames are interpreted and used by a receiver which can then more generally know the state of air traffic and act accordingly. The ADS-B receiver, an RTL-SDR USB dongle for example, decodes short frames (degraded mode) or long frames (full mode). At the end of the treatment, the short frames contain the international aircraft identifier (ICAO, International Civil Aviation Organization), the altitude of the aircraft and possibly its speed and direction of movement. The long frames also give the position (latitude—longitude) of the aircraft.

**Figure 1:**
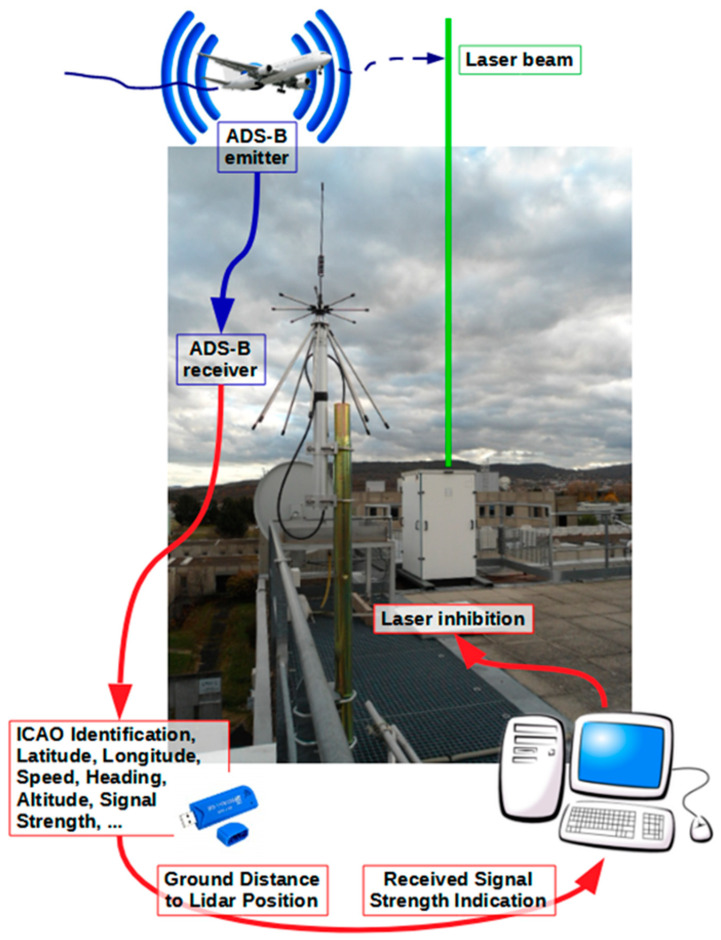
Overview of the air-safety system based on a picture of the outdoor OPGC platform.

Our safety method aims to suspend laser emission until the area is clear again by using free, open access software, an inexpensive ADS-B receiver, and the associated protocol (Figure 1).

### 2.3. Software Defined Radio Dongle Hardware

**Figure 2:**
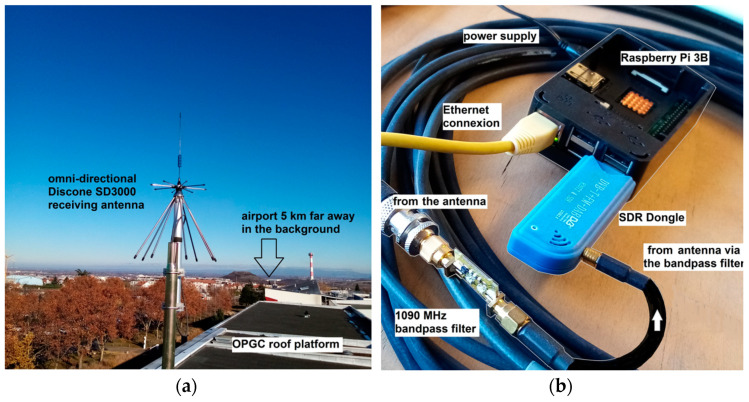
General view of the outdoor (**a**) and indoor (**b**) hardware with SDR Dongle and Raspberry Pi.

Several brands and models are marketed, but the USB RTL-SDR Tuner Receiver Dongle we use is of type RTL-SDR R820T2 (Figure 2, [10]). This model of RTL-SDR dongle, distributed on the internet, is equipped with a Realtek RTL2832U chipset and R820T2 tuner specially designed for SDR mode. It is a broadband receiver–scanner with a frequency range from 24 MHz to 1766 MHz in all modes including ADS-B (1090 MHz). We also improved the RF reception selectivity by adding a band-pass filter.

This dongle is supplied with a mini magnetic antenna but we prefer to use an omni-directional Discone SD3000 antenna. More generally, our receiving system is driven by a Raspberry Pi 3B, which is able to drive the dongle, process the data and transmit the alerts via an ethernet connection on the local network where the LIDAR computer can monitor them to switch off the laser.

### 2.4. Software

**Figure 3:**
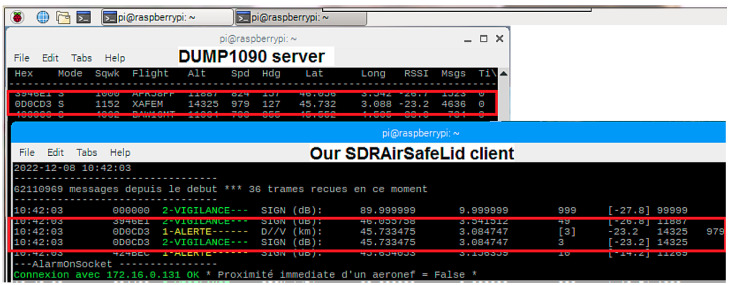
RaspberryPi screenshot illustrating software in use: DUMP1090 “server” (top) and SDRAirSafeLid “client” (bottom).

In this section we present an overview of the software devoted to managing the data acquisition (DUMP1090), the decision making (python-based “client” software, [11]) and the alert management. Incidentally, Virtual Radar freeware can help to visualize air traffic in real time [12]. A screenshot illustrating the software used is given in Figure 3.

#### 2.4.1. Data Acquisition

The data “server” software, DUMP1090, is a software that can be downloaded freely from GitHub [13]. This software provides the received signal strength and decodes the ADS-B frames to obtain, if available, the identifier of the aircraft, its altitude and its full coordinates (latitude, longitude, speed, direction).

We used the DUMP1090 freeware suite from GitHub. It was released under the BSD three-clause license. DUMP1090 was firstly written in 2012 by Salvatore Sanfilippo and then upgraded by Malcolm Robb. DUMP1090 is a simple MODE-S decoder specifically designed for RTL SDR devices. Its main features are robust decoding of weak messages, network support, embedded HTTP, single-bit error correction, the ability to decode the majority of DF messages and to decode raw IQ samples from files, an interactive command-line-interface mode where aircraft currently detected are shown in a list as more data arrives, CPR coordinate decoding and track calculation from velocity, TCP server streaming and raw data sharing capacity between connected clients (using—net). The acquired data are then used by the “client” software. 

#### 2.4.2. Analysis and Decision-Making

The python 2.7-based “client” software, “SDRAirSafeLid” [11], which was developed at the Technological Development Department of OPGC, uses a function “socket” which uses a low-level networking interface so we can read the data flow from the server and process this stream. An events management protocol has been developed considering the aircraft coordinates, received signal strength and other information such as altitude or knowledge of usual air traffic.

This software compares the proximity indicators received with predefined critical thresholds every second. This periodicity is compatible with the implementation of the required air safety. When the position of the aircraft is received, the ground distance separating the aircraft from the laser beam is calculated and compared to a critical distance fixed at 2 km. If the identity of the aircraft is transmitted but not its position, the strength of the received signal is then processed. This indicator is usually used in the field of telecommunications is the RSSI (Received Signal Strength Indication). Its value is always negative, given in dBm with 0 dBm when the theoretical signal is maximum and, in our case and in first approximation, inversely proportional to the proximity of the aircraft. The chosen threshold is fixed at −2.5 dBm. In the case of both ground distance and RSSI, if the alert threshold is exceeded, a logic command is sent to the LIDAR electronics to stop the laser emission. Scanning ADS-B frames continues during laser shutdown and laser emission can only resume when the required conditions are met again. The data used to signal an alarm and act on the laser emission are time-stamped and saved in two daily files, respectively, one called “VigiLog” for all data received and the other one, “AlarmLog”, for those that caused an interruption to the LIDAR activity. This ensures the traceability of events that required interruption of the laser. Usually, the lockouts last for a few seconds to a few minutes before the aircraft moves away from the critical area.

In summary, this system makes it possible to detect useful signals at a sufficient distance and therefore within sufficient time to exploit the information and to proceed to the inhibition of the laser so that the passage of the aircraft can meet the ocular safety requirements.

#### 2.4.3. ADS-B Alerts Management by COPLid

When an aircraft is detected by the ADS-B system, a byte is set to 1 and sent to the LIDAR supervisor software using a TCP/IP socket. As a server, the LIDAR PC receives this byte from the ADS-B system hosted on a Raspberry Pi connected to the LAN of the OPGC. This causes the LIDAR supervisor software to activate an alarm state. In this state, the laser stops emitting any radiation. As long as the byte is set, the supervisor remains in the alarm state. When the ADS-B system resets to 0 for the alarm byte, the LIDAR PC acknowledges it. Then the LIDAR supervisor leaves the alarm state and enters a temporization state for 20 s. This delay is necessary to avoid multiple stops and restarts of the laser caused by an individual ADS-B alert; for instance, when the trajectory of an aircraft cuts the warning perimeter several times or when the alert is generated by the strength of the received signal. Then, after leaving the temporization state, the software restarts the laser emission without acquisition, because a period of warmup time of the laser is necessary in order to record the backscattered light signal when the laser energy is stable. As the laser does not completely stop during an ADS-B alarm, because only the laser cavity is blocked and the lamp keeps on flashing without any emission of laser radiation, the warmup time is reduced to 30 s before restarting the acquisition.

## 3. Results and Discussion

### 3.1. Air Traffic Aspects of the Site

**Figure 4:**
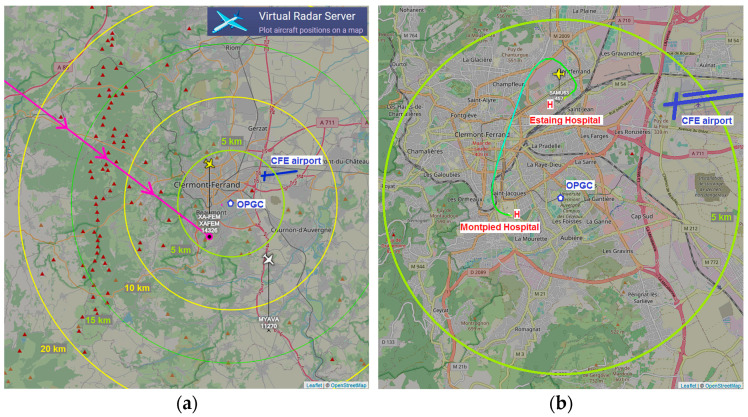
Screenshots from Virtual Radar Server based on OpenStreetMap showing an example of international flight (XA-FEM, Servicios Aereos Regiomontanos SA) (**a**) and of local flight (SAMU63, medical emergency helicopter) (**b**). Concentric yellow and green circles give the ground distance to the COPLid location.

COPLid is located on the Cézeaux University campus in Aubière (45°45.68′ N, 3°6.65′ E) and is installed on the OPGC platform, which is less than 5 km from two Clermont Ferrand hospitals (Estaing and Montpied) and CFE airport (Figure 4). 

The French Aeronautical Information Service (SIA) provides information on the visual approach and landing plans that shows that the approach corridors of Clermont-Ferrand airport (CFE) do not pass directly over the OPGC. The national or international air traffic in transit is also mainly confined to corridors away from our position. One of the objectives of our tests was to confirm this information and characterize the marginal air traffic that may interact with our scientific activities. For this, we have recorded all the events that would require a shutdown of our laser in case of normal scientific exploitation.

### 3.2. Risk Assessment for Air Traffic

The high-power laser beam is emitted vertically, which means that an aircraft or a helicopter cannot pass through the beam at an eye-safe distance. Due to the pointing of the laser radiation towards the zenith, the essential risk is ocular risk for the pilot, either in the case of direct vision towards the source, at nadir, or in the case of the presence of a reflector that allows vision at nadir. In order to provide a reliable security system, it is first necessary to characterize the type of air traffic events and the corresponding risks according to increasing probability of occurrence.

Distant aircraft that do not pass in immediate proximity to the axis of the laser beam are not affected by the radiation. In the case of these aircraft, and although the risk is null, our security system will take preventive care regarding this type of air traffic within a radius of several kilometers around the LIDAR. This will make it possible to know the behavior of aircraft that could eventually become critical on a later approach.

Figure 4 presents two examples of events having triggered the shutdown of the laser emission. Figure 4a represents a typical international transit with the SAR SA flight operating at an altitude of 14,326 m, at a speed of 980 km.h^−1^. The aircraft is a Gulfstream G600. In this case, it is physically impossible for the pilot to have visual contact directly with the laser source at nadir. Furthermore, the risk of an indirect reflection of the light source into the eye seems unlikely. An example of this would be if the cabin of an aircraft was positioned vertically to the laser returning the beam very precisely toward another aircraft whose pilot would be looking in that direction at the same time. Although the risk is extremely unlikely for this type of traffic and is only based on a precautionary principle, the laser emission will be interrupted by our security system.

Figure 4b represents a typical local flight in SAMU63 at an altitude of 457 m. This medical emergency helicopter typically lands on the Estaing Hospital heliport after passing approximatively 2 km west of OPGC and having initially come from the Gabriel Montpied Hospital. In the case of a helicopter in local transit or that of an aircraft at low altitude in an approach maneuver, the probability of visual contact with the nadir for the pilot is not null. In this situation, the ocular risk is confirmed when the aircraft flies over the LIDAR in operation. For this type of traffic, the security system that we implement will eliminate this potential risk by stopping the laser emission. To consider the ocular risk described, whatever the probability or mode of occurrence, we have set up a device which effectively ensures the temporary extinction of the laser.

### 3.3. Impact of the Air Traffic on the Temporal Coverage of LIDAR Operation

COPLid supplies continuous aerosol measurement to the European scientific community in the frame of ACTRIS Research infrastructure (Aerosols Clouds Trace gazes Research Infra-Structure, [14]). LIDAR produces data profiles every 60 s which corresponds to 600 laser shots. Vertical raw profiles are stored in a raw data file. The files sent to ACTRIS SCC (Single Calculus Chain, [15]) aggregate one hour of measurement in one NetCDF file, which means 36,000 laser shots (or 60 raw data files) in the best case scenario. Moreover, the duration of all the raw data files should be the same with a tolerance of 10%, which means between 54 and 66 s in the COPLid case. Consequently, longer files must be excluded from the NetCDF submission file, which means those data are lost.

In this context, optimization of the ADS-B alerts is very important because the suspension of the LIDAR acquisition may cause a non-negligible lack of laser shots. Each ADS-B alert provokes a minimum interruption of 50 s, which covers the temporization and warmup times. Therefore, the minimum lack of shots is 500. However, it is also necessary to consider that when an alert occurs, the recorded file in progress is destined to be lost. Indeed, its duration would be much more than 60 s if we decided to resume the acquisition after the alert: it would be at least 60 s (acquisition time) and 50 s (temporization + warmup), which means at least 110 s. As mentioned above, a file exceeding 66 s must not be submitted to the SCC because it would cause a processing error. As we take into account that a record in progress must be rejected when an ADS-B alert occurs, the average time of lost data due to canceled files is 30 s considering that an ADS-B event can occur with the same probability within a 60 s recording. All this means that when an ADS-B alert occurs, we assume that the minimum average time of lost data is actually 50 s of temporization and warmup plus a 30 s average lost record. Therefore, each isolated ADS-B alert provokes an average of 80 s of lost data, plus the duration of the alert itself.

**Figure 5:**
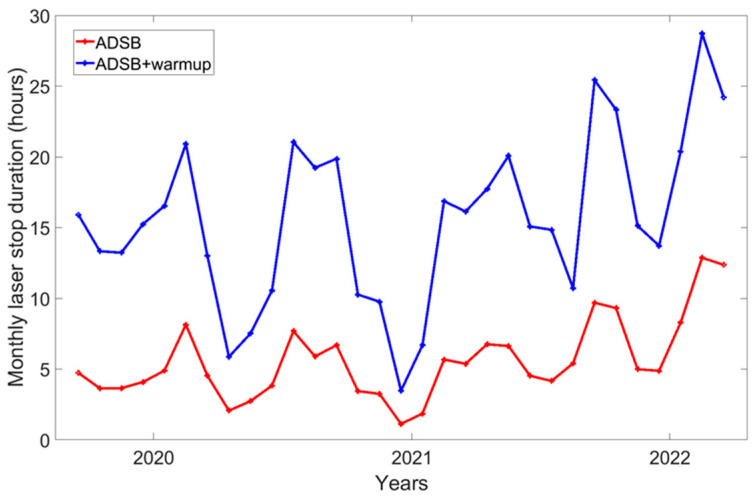
Monthly laser stop and measurement durations. The red line corresponds to the monthly laser stop duration, and the blue line to the measurement stop duration, also including the temporization and warmup time needed to stabilize the laser emission.

**Figure 6:**
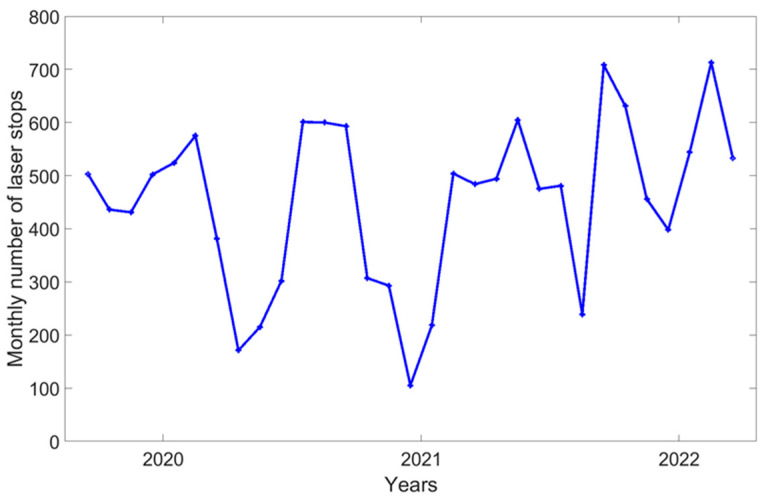
Monthly number of laser stops.

An air traffic detection data set was acquired during the two years 2020 and 2021. The security system operated continuously under normal conditions of the LIDAR. Thanks to the log files of ADS-B events continuously recorded since 2020, it has been possible to assess the monthly and daily duration of lost data (Figure 5 and Figure 6). The mean duration of ADS-B alerts is 6 h a month and 702 s a day.

The average duration per day of ADS-B alerts is spread over an average of 15 events per day which means 15 warmup times to add (50 s) and 15 data files lost with an average time of 30 s.

This means that the daily average duration of data loss is 702 s + (15 × 80 s) = 1902 s. The hourly average impact on the air traffic is a lack of 79.25 s/hour, which means the loss of 793 laser shots out of 36,000 or a 2% missing data rate. The study period (2020–2021) corresponds to the COVID-19 pandemic [16]. During this period, there were fewer aircraft in flight [17] and therefore fewer aircraft detected by the system. Signatures of reductions in air traffic during the lockdowns are visible in Figure 5 and Figure 6 with low values for the number of laser stops and their duration in April–May 2020 and December 2020. A longer study period is necessary to more precisely evaluate the impact of the air traffic on LIDAR operation.

When an airline passes over Clermont-Ferrand, an alert may last for a few seconds, which leads to a loss of about one raw datafile, corresponding to 600 laser shots. However, when a helicopter lands on a drop zone inside the safety perimeter, typically at the Gabriel Montpied Hospital in the case of COPLid, the alert event may last for several minutes. If such events occur several times in the same hour, it might be possible that the number of missing laser shots is greater than 18,000 shots, which would make data submission impossible for this measurement hour. To avoid this scenario, the algorithm of the ADS-B system has been modified to skip alerts triggered by a helicopter landing at a safe distance away. The phases of helicopters landing, standing by and taking off are identified based on a combination of the helicopters’ known localization, altitude and speed. For example, even if the hospital heliport is only one kilometer away from the LIDAR site, this allows us to define these aircraft attitudes as safe and it does not require us to stop the laser.

This modification was achieved very recently, in summer 2022. For this reason, we do not yet have a sufficient data set of ADS-B events to evaluate the impact of this modification on the LIDAR measurement.

The security system that we have developed can evolve by considering a logic of processing integrating parameters such as, for example, prior knowledge of local air traffic (known regular traffic), altitude, speed, direction or anticipation of an approach maneuver by the aircraft. Alert threshold parameters also contribute to the flexibility of our system according to the constraints of any additional features such as traffic or local topology.

## 4. Conclusions

The system described in this article successfully passed the development tests. Our method allowed us to identify aircraft detected in other ways: either visually, audibly or by referring to a flight-tracker website [18]. We can also identify aircraft such as some military ones, which are not referenced publicly on such websites. Currently, detected aircraft within a radius of several tens of kilometers around the LIDAR are considered. LIDAR operation is not disturbed by the presence of birds or UAVs (Unmanned Aerial Vehicle). The frames from tens of aircraft can be received and processed at the same time. Even in degraded mode, without having the exact position of the aircraft, the safety procedure is successfully applied by processing the strength of the received signal (RSSI). 

Our system processes the available ADS-B information from nearby aircraft and, if needed, stops laser emission with enough efficiency that the current aviation safety requirements are met. Consequently, in accordance with the evolution of air safety rules, the OPGC site has been successfully accredited by the French air authorities, the “Direction Générale de l’Aviation Civile” (DGAC). 

Concerning LIDAR data quality control, the rate of missing data caused by air traffic interruption is about 2%. This is a satisfactory result with regard to the data temporal coverage requested by observation networks such as ACTRIS. We are still working to improve our system. For example, this rate has been recently lowered by improving the algorithm so that it considers the specificity of helicopters (local traffic). 

The effectiveness of our air safety procedure helps us to operate daily outdoor lasers efficiently and safely. In the future, this cost-saving system can be deployed for permanent activity of LIDAR in other observatories, such as those that are part of the ACTRIS network. For successful accreditation, special attention should be paid to the type of air traffic in the vicinity. This system has already been successfully tested but is not yet accredited on different sites such as OPAR [19] at La Réunion Island or LOA at Lille. It could also be used effectively alongside portable LIDARs during field campaigns. 

## Data Availability

The data presented in this study are available at https://github.com/FredPey/SDRAirSafeLid (accessed on 6 March 2023).
